# Achieving the UNAIDS 90–90-90 targets: a comparative analysis of four large community randomised trials delivering universal testing and treatment to reduce HIV transmission in sub-Saharan Africa

**DOI:** 10.1186/s12889-022-14713-5

**Published:** 2022-12-13

**Authors:** K. Sabapathy, L. Balzer, J. Larmarange, L. Block, S. Floyd, C. Iwuji, K. Wirth, H. Ayles, S. Fidler, M. Kamya, M. Petersen, D. Havlir, F. Dabis, J. Moore, R. Hayes

**Affiliations:** 1 London School of Hygiene and Tropical Medicine, London, UK; 2 University of California Berkeley, Berkeley, USA; 3 Centre Population et Développement, Université Paris Cité & Institut de Recherche pour le Développement, Inserm, Paris, France; 4 Centers for Disease Control & Prevention, Atlanta, USA; 5 Peraton, Atlanta, USA; 6 Africa Health Research Institute, Durban, KwaZulu-Natal South Africa; 7 Brighton and Sussex Medical School, Brighton, UK; 8 Harvard School of Public Health, Boston, MA USA; 9 Zambart, Lusaka, Zambia; 10 Imperial College London, London, UK; 11 Infectious Disease Research Collaboration, Kampala, Uganda; 12 Makerere University, Kampala, Uganda; 13 University of California San Francisco, San Francisco, USA; 14 Bordeaux University, Bordeaux, France

**Keywords:** HIV, Antiretroviral treatment, Universal Testing and Treatment, Treatment as Prevention, UNAIDS 90-90-90

## Abstract

**Background:**

Four large community-randomized trials examining universal testing and treatment (UTT) to reduce HIV transmission were conducted between 2012–2018 in Botswana, Kenya, Uganda, Zambia and South Africa. In 2014, the UNAIDS 90–90-90 targets were adopted as a useful metric to monitor coverage. We systematically review the approaches used by the trials to measure intervention delivery, and estimate coverage against the 90–90-90 targets. We aim to provide in-depth understanding of the background contexts and complexities that affect estimation of population-level coverage related to the 90–90-90 targets.

**Methods:**

Estimates were based predominantly on “process” data obtained during delivery of the interventions which included a combination of home-based and community-based services. Cascade coverage data included routine electronic health records, self-reported data, survey data, and active ascertainment of HIV viral load measurements in the field.

**Results:**

The estimated total adult populations of trial intervention communities included in this study ranged from 4,290 (TasP) to 142,250 (Zambian PopART Arm-B). The estimated total numbers of PLHIV ranged from 1,283 (TasP) to 20,541 (Zambian PopART Arm-B). By the end of intervention delivery, the first-90 target (knowledge of HIV status among all PLHIV) was met by all the trials (89.2%-94.0%). Three of the four trials also achieved the second- and third-90 targets, and viral suppression in BCPP and SEARCH exceeded the UNAIDS target of 73%, while viral suppression in the Zambian PopART Arm-A and B communities was within a small margin (~ 3%) of the target.

**Conclusions:**

All four UTT trials aimed to implement wide-scale testing and treatment for HIV prevention at population level and showed substantial increases in testing and treatment for HIV in the intervention communities. This study has not uncovered any one estimation approach which is superior, rather that several approaches are available and researchers or policy makers seeking to measure coverage should reflect on background contexts and complexities that affect estimation of population-level coverage in their specific settings.

All four trials surpassed UNAIDS targets for universal testing in their intervention communities ahead of the 2020 milestone. All but one of the trials also achieved the 90–90 targets for treatment and viral suppression. UTT is a realistic option to achieve 95–95-95 by 2030 and fast-track the end of the HIV epidemic.

**Supplementary Information:**

The online version contains supplementary material available at 10.1186/s12889-022-14713-5.

## Introduction

### UNAIDS 90–90-90 targets

UNAIDS set targets in 2014 for 90% of people living with HIV (PLHIV) to be diagnosed and aware of their HIV-positive status, 90% of these to receive antiretroviral treatment (ART) and 90% of these to be virally suppressed by the year 2020 [1]. UNAIDS data indicate that an estimated 26 million PLHIV globally were receiving ART in 2020, representing ~ 67% of the 38 million PLHIV and approximately 4 million below target [2]. Modelling suggests that there were 3.5 million more new infections and 820,000 more deaths from HIV between 2015 and 2020, than if 90–90-90 targets had been met by 2020 [2].

Ambitions to “fast track” the end of the epidemic continue and UNAIDS targets for 2030 are to go beyond 90–90-90 to achieve 95–95-95 [3]. The concept of using extensive treatment coverage to achieve reductions in circulating virus, reduce transmission of HIV and therefore reduce HIV-incidence gained widespread interest in 2009 following a study which modelled universal testing and treatment to reduce population HIV viral load as a prevention approach [4].

### UTT trials to deliver treatment as prevention

Four large community randomized trials examining universal testing and treatment (UTT) to reduce HIV transmission and improve health outcomes were conducted between 2012–2018 [5–8]. The Ya Tsie Botswana Combination Prevention Project (BCPP) trial was conducted in Botswana, the HPTN 071 Population Antiretroviral therapy to Reduce Transmission (PopART) trial in Zambia and South Africa (Western Cape), the Sustainable East Africa Research in Community Health (SEARCH) trial in Kenya and Uganda, and the ANRS 12,249 Treatment as Prevention (TasP) trial in South Africa (Kwa Zulu Natal).

The trials were designed before the UNAIDS 90–90-90 targets were set, but had the aim of optimising coverage and the 90–90-90 targets have since been adopted as a useful metric to monitor coverage. The mainstay of HIV testing and linkage to care in the TasP, BCPP and PopART trials was door-to-door HIV services provided by community health workers. The PopART trial had two intervention arms, in Arm A door-to-door HIV testing was combined with immediate eligibility for ART from the start of the trial, while in Arm B eligibility for ART was according to national guidelines (initially with CD4 cut-off of 350c/mm^3^ and 500 c/mm^3^, later transitioning to immediate eligibility when WHO guidelines changed). The SEARCH trial used a hybrid model of multi-disease community-based health fairs (for health education; screenings for HIV and diseases like hypertension, diabetes, and tuberculosis; and immediate care or referral for any health problems) and mobile outreach. All the trials also used a range of additional approaches to support rapid ART start and viral suppression among persons who started ART [9–14]. Some examples include—linkage to treatment services including appointment-scheduling, SMS appointment reminders, and tracing activities for those who missed initial appointments (BCPP); CHiP-assisted linkage to clinic (PopART); initiation of same day ART (SEARCH); dedicated local HIV clinics and linkage to care team (TasP).

Achievement of high coverage across each step of the HIV care cascade was a key mechanism by which the intervention package in each of the UTT trials aimed to reduce HIV incidence and improve community health outcomes.

### Comparative analysis of the UTT trials

The overarching hypothesis behind UTT as a public health approach to prevent HIV is that if universal coverage as measured by high levels of population viral suppression is achieved, prevention at a population level will be realised [4, 15]. The designs of the trials, individual trial methods used to estimate coverage and the extent to which coverage estimates reflect the HIV-incidence, morbidity and mortality reductions in the trial intervention communities have been described elsewhere [6, 8–13, 16–18].

Here we systematically review the approaches used by the trials to measure intervention delivery, and estimate coverage—largely driven by different contexts and measurement schemes—against the 90–90-90 targets. We aim to provide an in-depth understanding of the background contexts and complexities which affect estimation of population level coverage of the steps of the cascade related to the 90–90-90 targets, by comparing and contrasting the approaches used.

## Methods

### Data assimilation for comparative analysis

Trials summarised the methods used by their respective trial to estimate coverage and shared data used for estimation of 90–90-90 cascade coverage targets and population-level viral suppression. The principles espoused by the PRISMA statement [19] were adapted and used to examine the basis for the 90–90-90 coverage estimates in each trial. We explored trial-specific approaches to estimating the numerators and denominators for each of the 90–90-90 coverage measures at baseline of the trial (prior to delivery of trial interventions) and at endline – defined as the conclusion of delivery of trial interventions. We present the primary approaches taken to estimate coverage, although each trial implemented several secondary and sensitivity analyses which are described elsewhere [6, 8–14, 16–18]. We also present each trial’s 90–90-90 coverage measures, overall and by sex, so that coverage achieved by each trial can be compared, with due regard to the different approaches used. The potential for bias was actively explored and is discussed below. We also highlight the strengths and limitations of the approaches used to enable policy makers and researchers to adapt methods used by the UTT trials for future implementation.

Ethical approval was not required for this paper as we report only previously published data. Ethics approval was granted for the respective trials from the relevant ethics committees [10–13].

### Analysis of population and data sources

We focus on cascade coverage achieved in intervention communities at study baseline and endline and estimates were predominantly based on “process” data obtained during the process of delivering the intervention. Only trial communities with accurate data at both baseline and endline were included (Table 1), therefore excluding PopART South African intervention communities where there were recognised inaccuracies in baseline data collection. Further, to enhance comparability across trials only communities which received the intervention for a duration of at least ~ 30 months were included (5 out of 11 intervention communities in TasP), therefore excluding those TasP intervention communities which were added to the trial later and had shorter total periods of follow-up.

**Table 1:** 90–90-90 related key information

	**BCPP**	**PopART**	**SEARCH**	**ANRS 12,249 TasP**
Number of intervention communities included in this analysis	- 15 communities	- 4 arm A and 4 arm B Zambian communities^a^	- 16 communities	- 5 communities with at least ~ 30 months of follow-up^a^
Average trial community size	- 5,785	- 44,000	- 10,450	- 1,284
Estimated HIV prevalence in communities at baseline	- 29%	- Average ~ 15% (range 10–25%)	- Average 11% (range 2–25%)	- ~ 29%
Eligibility criteria for being offered the trial intervention	- Age 16-64y - On average ≥ 3 nights per month and more nights in the household than any other in the community over preceding 12 months - Documented citizenship of Botswana or marriage to citizen	- Age ≥ 18y baseline and lowered to ≥ 15y at endline - Participant defined residence in household in trial community - No restriction based on nationality	- Age ≥ 15y - Participant defined residence in household in trial community - No restriction based on nationality	- Age ≥ 16y - Resident (for at least 4 nights /week within homestead in general) - No restriction based on nationality
Intervention observation period included in this study	- ~ 30 months from baseline to endline intervention round (inclusive of information obtained at endline)	- ~ 48 months from baseline to endline intervention round (inclusive of information obtained at endline)	- ~ 36 months from baseline to *before* endline intervention round	- ~ 30 months from baseline to endline intervention round (inclusive of information obtained at endline)
Approach to measuring denominators and numerators for 90–90-90	Total population and HIV prevalence estimation: - Fixed representative sample identified at baseline—simple random 20% sample of households in the community at baseline (assumed unchanged at endline; no repeat census /updated estimate at endline) - HIV prevalence in study eligible population in each community standardized to the 2011 Botswana Census by age and sex Knowledge of HIV status, ART uptake and viral load data: - Obtained from population present at time of intervention round - Updated during each intervention round (approximately annually)	Total population, HIV prevalence, knowledge of HIV status and ART uptake data: - Total population present at time of intervention round - Community-wide household census with baseline intervention round - Updated during each intervention round (approximately annually) Viral load data: - Random sample of 75 HIV positive individuals selected from *Population Cohort*^b^ at baseline and endline	Total population, HIV prevalence, knowledge of HIV status, ART uptake and viral load data: - Total population present just prior to intervention round - Community-wide household census at baseline and at endline (three years later)	Total population, HIV prevalence, knowledge of HIV status, ART uptake and viral load data: - Total population present during community-wide household census at time of intervention round - Updated during each intervention round (approximately every 6 months)
Sources of data collected during intervention	- Knowledge of HIV status, ART uptake and viral load data obtained from electronic heath records or patient held documentation	- Knowledge of HIV status and ART uptake data obtained through self-report during delivery of intervention - Viral load data obtained through venous blood samples from *Population Cohort*^b^ obtained for research purposes	- Knowledge of HIV status, and ART uptake data obtained from electronic heath records - HIV status and viral load data obtained during population-level community based testing (multi-disease health fairs with home-based (or other location of choice) testing for persons not attending the health fair)	- Knowledge of HIV status, ART uptake and viral load data obtained from electronic heath records^c^

^a^PopART – only Zambian communities are examined in this paper due to problems in data collection at baseline of intervention delivery in the South African PopART communities; TasP—six intervention communities with only 4 survey rounds (~ 18 months of follow-up) were excluded in this paper to allow better comparability with other trials with ≥ 30 months of follow-up. For the purpose of this paper “endline” in TasP is considered the sixth intervention round

^b^*Population Cohort* = Random sample of 2000–2500 individuals from each community sampled to provide research questionnaire data and blood samples for information on HIV status, viral load etc.

^c^If a HIV-negative status was documented before a given home-visit and a positive status after, date of seroconversion was imputed using a random point approach (uniform distribution); For those observed HIV negative before a given home-visit but with no subsequent HIV status, imputation of possible unobserved seroconversion using observed incidence by sex and cluster

There were a few exceptions to the use of process data. In the BCPP trial a cohort of residents were enrolled from a random 20% sample of households in the participating communities to assess pre-specified baseline and endline outcomes, including those relevant for the 90–90-90 targets. Household and individual level data from this sample were used to estimate the total eligible population and the estimated number of HIV positive persons living in the 15 intervention communities. Additionally, in PopART, all coverage related data were process data obtained through intervention delivery except viral load measures which were extrapolated from the main trial research cohort – the *Population Cohort*. The *Population Cohort* consisted of randomly sampled individuals from each trial community and viral load testing was carried out on a random subset of HIV-positive *Population Cohort* members at baseline and another random subset of HIV-positive *Population Cohort* members at endline.

The number of intervention communities per trial examined in this paper ranged from five to 16 (Table 1). Baseline estimates of HIV prevalence in these communities ranged from 10–29% across the trials [9]. Age eligibility criteria for inclusion in the intervention varied across the trials. Cascade coverage estimates presented here use a lower age limit of 15–16 years. BCPP further included an upper age limit of 64 years. In keeping with national policies related to access to free healthcare, only citizens of Botswana were included in the BCPP study, but none of the other studies were required to apply restrictions based on nationality.

The time-interval for data collection from the start of intervention delivery to the end of follow-up varied from 30–48 months. In SEARCH, 90–90-90 related information at endline refers to coverage prior to the final delivery of trial intervention services, while endline information in the other three trials accounts for services delivered during the final round of intervention delivery.

### Population enumeration

At a population level, identifying the total number of PLHIV would ideally rely on assessment of HIV status among all eligible members of the community, or among a representative sample (or equivalently, an accurate estimate of HIV prevalence and population size). A key first step in this process is thus enumeration of the eligible population resident in each intervention community at baseline and endline.

TasP, PopART and SEARCH conducted population-wide surveys at baseline and endline to determine information on who was living in the community at a given time-point (including age and gender of inhabitants if the information was obtainable) and account for in- and out-migration and deaths by endline to be captured. PopART and TasP did this with each round of intervention delivery in the communities (PopART annually and TasP six monthly), while SEARCH conducted surveys just prior to intervention delivery at baseline and at endline.

In BCPP, the most recent Google satellite imagery available prior to study start was used to identify all plots with household-like structures for each trial community at baseline. From a list of household-like structures, a 20% random sample of plots were selected for enumeration and enrolment from each community. The proportion of plots with residential, habitable, and regularly-occupied households was determined and among regularly-occupied households consenting to enumeration, the average number and age and sex distribution of study-eligible household members were obtained. These data were then extrapolated to the remaining 80% of plots with household-like structures to estimate the total number of study-eligible residents in each community at baseline, stratified by age and sex. The same baseline estimate was used for the total population at end-line, with the assumption that in- and out-migration were negligible between baseline and endline.

### Total PLHIV

In SEARCH, the primary source of data at baseline was rapid HIV antibody testing conducted at community health fairs, as well as at home or other location of choice for persons not attending the health fair (Table 1 and Supplementary Table 1). SEARCH further adjusted for differences in the characteristics of persons participating in health fairs and home-based testing as compared with the enumerated population, thereby extrapolating results to non-participants. To do so, within each community separately, they used testing data to estimate HIV prevalence within values of baseline covariates (e.g. age, sex, occupation, education, mobility) and then standardized estimates with respect to the community-specific covariate distribution [20]. In PopART, the primary source of data at baseline was rapid HIV antibody testing conducted at the household and information on self-reported prior knowledge of HIV-positive status was further used among those not testing at the household. HIV test results among intervention participants who accepted testing were used to estimate HIV prevalence among participants who did not already know they were HIV-positive but did not accept testing, and following this the total PLHIV among participants was estimated. HIV prevalence among participants was then extrapolated to non-participants, to estimate the total PLHIV in the community.

TasP accessed routine health records to identify individuals previously diagnosed with HIV prior to baseline and they added information from data obtained in trial delivered clinics. Further, dried blood sampling for HIV viral load was done as part of the trial intervention in the community (all individuals were asked to provide a sample at the household irrespective of HIV status). This could include individuals who declined HIV testing and were not diagnosed with HIV but who agreed to provide a dried blood spot sample for viral load measurement for research purposes. If a negative status was documented before a given date of contact with an individual (t) and a positive status after t, date of seroconversion was imputed using a random point approach (uniform distribution). For those observed HIV negative before t but with no subsequent HIV status after t, imputation of possible unobserved seroconversion was done based on observed incidence by sex and cluster. Those with no observed HIV status were excluded from analyses.

For BCPP, age- and sex-stratified HIV prevalence at baseline of each community was derived from the 20% random household sample. The community-specific stratified estimates were then standardized to the community-specific age and sex distribution reported by the 2011 Botswana Census. The estimated total number of HIV-positive persons by age and sex in each community at baseline was calculated by multiplying the community-specific study eligible residents and adjusted HIV prevalence by age and sex.

The total PLHIV at endline in BCPP was assumed to be unchanged from baseline. For the other trials at endline, the same approach used at baseline was applied additionally extending to i) account for persons already known from baseline testing to be positive; and ii) to further adjust for prior testing history (intervention participation) in addition to baseline covariates. In SEARCH, estimates of population HIV prevalence were obtained with targeted maximum likelihood estimation (TMLE) and converted to a number of PLHIV using estimates of the total population size [20]. Similarly, PopART stratified on community, age group and sex but at endline also on prior residency and prior participation in the intervention and HIV self-report and testing history.

### PLHIV with knowledge of HIV-positive status

For the baseline measure of knowledge of HIV status all the trials used knowledge prior to testing offered by the trials. At endline, all the trials except SEARCH included knowledge of HIV status obtained from the endline provision of testing. In SEARCH endline knowledge was that just prior to the final health fair. All the trials except PopART used data from existing routine health records for knowledge of HIV-positive status at baseline. PopART used self-report verified by patient held records if possible, while BCPP and TasP used self-report verified by patient held records as an additional source of information to that obtainable by linking to electronic heath records.

In BCPP, TasP, and SEARCH prior documentation of ART uptake was included as evidence of prior knowledge of HIV-positive status, while SEARCH further used an undetectable viral load, obtained at the health fair (or traced subsequently), for the same. In TasP, BCPP and SEARCH, comprehensive coverage of routine health care records in the area and, in the case of TasP, trial-run clinics delivering clinical care, meant that near-completeness of observed data was assumed. In these trials, therefore, persons without a record of a prior positive test or ART use (as well as, for SEARCH, without a suppressed viral load) were considered to have unknown status. In PopART, information on self-reported prior knowledge of HIV-positive status was collected during the baseline of intervention delivery without using routine records. Because PopART relied primarily on self-report when assessing HIV status, measures were only obtained from persons agreeing to participate in the intervention. Therefore, PopART used methods analogous to those used to estimate number of PLHIV to extrapolate knowledge of HIV status to the larger population, adjusting for differences between participants on whom self-reported knowledge of status was measured and non-participants for which it was not.

### PLHIV on ART

In BCPP, pre-existing documentation of being on ART (ART cards, pill bottles and electronic medical records) was used to identify those already receiving ART at baseline. In PopART self-report was used with verification from patient-held ART cards if possible. SEARCH and TasP both used routine health records of prior ART initiation. SEARCH also used undetectable viral load, measured during population level testing at a health fair (or traced subsequently), as evidence of prior treatment with ART, while TasP used an undetectable VL in routine electronic clinical care records in the 13 months prior to the first round of intervention delivery as evidence of prior treatment (even if ART was not reported or documented).

SEARCH, PopART and TasP used approaches analogous to their baseline method for measuring the number on ART at endline. For SEARCH this again reflected status prior to the last round of intervention delivery, and for PopART, TasP and BCPP it included final intervention delivery. TasP additionally included ART initiation in trial or Ministry of health clinic within three months after last home visit for intervention delivery. In BCPP, the number of people on ART at endline was measured from electronic record of ART refill within the previous four months; a clinic appointment within the previous six months after a prior ART initiation date; or status as “on HAART/on therapy” in the electronic record. The presence of a viral load result in the previous 18 months after initiation was also evidence of retention on ART (the vast majority had results in the previous six months but due to realities of routine practice and possibility for missed results etc., the time period allowed for viral load testing was extended up to 18 months prior) in BCPP.

As for knowledge of HIV status, because PopART relied primarily on self-report when assessing ART use, measures were only obtained from persons agreeing to participate in the intervention. Therefore, PopART used methods analogous to those used to estimate PLHIV to extrapolate to the larger population, adjusting for differences between participants on whom ART status was measured and non-participants. In contrast, the other three trials relied on comprehensive capture of ART use from clinical records, which did not rely on active intervention participation, and thus classified PLHIV without ART documentation (and/or viral suppression) as not on ART.

### PLHIV on ART with viral suppression

In TasP, viral load data were obtained from trial clinics or routine electronic clinical care records (viral suppression defined as < 400 copies/mm^3^). Additionally, linear interpolation was performed between time points, and if there was no viral load prior to the baseline intervention related home-visit for a given individual, it was assumed they were not virally suppressed.

In BCPP, viral load data (with suppression defined as < 400 copies/mm^3^) were not collected as part of the baseline interventionbut were obtained from routine health records of PLHIV at endline. Although not included here, viral load measurements were obtained among a random sample of participants at baseline as part of the evaluation cohort and these data are reported elsewhere [18].

SEARCH used viral load measures collected at a population level at health fairs (or traced subsequently) to measure viral suppression (< 500 copies/mm^3^) at both baseline and endline. SEARCH further extrapolated results to PLHIV without a directly measured viral load, assuming that within values of baseline characteristics (e.g. age, sex, occupation, education, mobility), viral suppression among those with a measured viral load at health-fairs or (or traced subsequently) was representative of suppression among those not measured [20]. Again, estimates of population coverage were based on targeted maximum likelihood estimation (TMLE).

Viral suppression (< 50 copies/mm^3^) in PopART was measured in a random subset of ~ 75 PLHIV in each community, who were participants in the *Population Cohort (PC).* The average proportions virally suppressed in Arm A and Arm B communities were weighted according to the age-sex distribution of the HIV-positive population; applying PC HIV prevalence estimates to the final intervention data on age/sex structure of the population. However, despite the random sampling approach of the *PC,* in practise the majority of participants in the PC were female and 77–95% of those who had viral load testing were women, so that comparisons by gender are not meaningful to present.

For endline all the trials used an analogous approach to baseline, with the inclusion of data from prior intervention participation and care history (e.g. prior testing and suppression in the estimation approach for SEARCH).

## Results

### Total population and total PLHIV

**Fig. 1:**
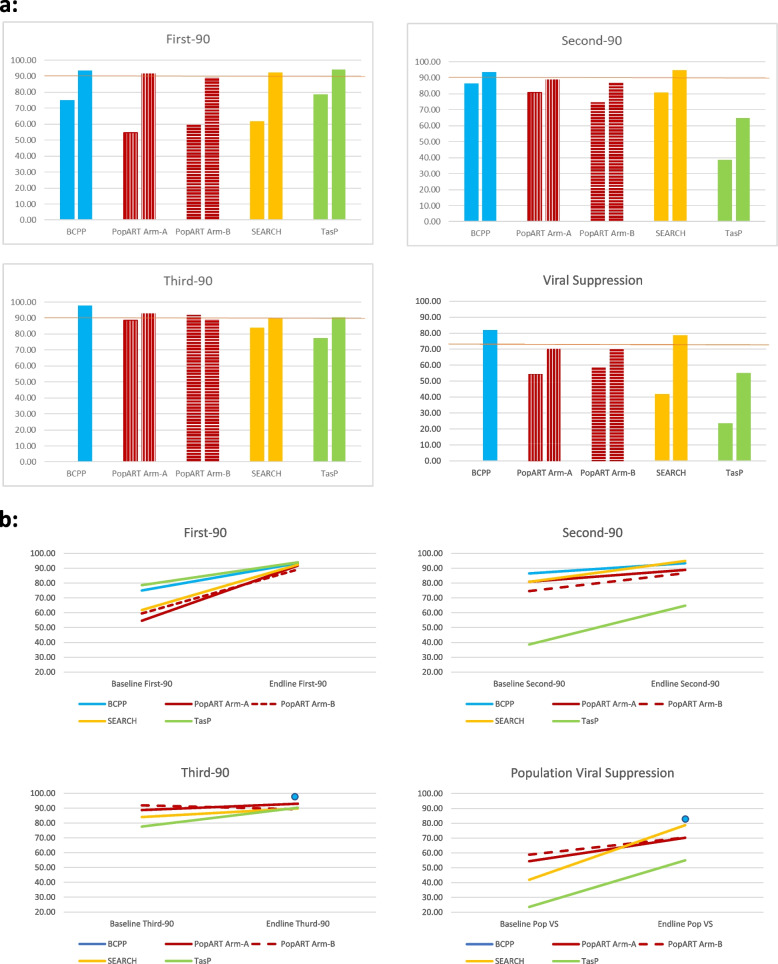
**a** Baseline vs Endline 90–90-90 and viral suppression estimates. **b** Differences made to 90–90-90 and population viral suppression estimates from baseline to endline

In BCPP through household enumeration and extrapolation to households that were inhabited but were not be enumerated, 61,544 study eligible individuals were estimated to reside in the 15 intervention communities (Table 2 and Fig. 1a). Approximately 14,270 HIV-positive individuals were estimated to reside in the 15 communities. However, 64,086 individuals were encountered and 61,655 had their HIV status assessed (2,431 (3.8%) of individuals interviewed refused to have their HIV status assessed).

**Table 2:** Baseline total population 90–90-90 coverage estimates

	**BCPP**		**PopART**				**SEARCH**		**TasP**	
	N	%	A (N)	A (%)	B (N)	B (%)	N	%	N	%
**Estimated total population**	61,544 (64,086)^a^	100%	125,911	100%	134,839	100%	79,818	100%	4681	100%
**Total number with HIV status ascertained** **(+ ve and -ve)**	(61,655)^a^	(96.2%)^a^	83,487	66.3%	82,269	61.0%	71,402	89.5%	4024	86.0
**Estimated total HIV + **	14,270	23.1%	17,686	21.2%	18,274	22.2%	8305	10.4%	1159	28.8
**Knowledge of HIV + (First 90)**	10,703	75.0%	9648	54.6%	10,877	59.5%	5136	61.8%	911	78.6%
**On ART (Second 90)**	9258	86.5%	7805	80.9%	8112	74.6%	4149	80.8%	352	38.6%
**Virally suppressed (3**^**rd**^** 90)**	NA	NA	Sampled from *PC*^b^	88.7%	Sampled from *PC*^b^	91.9%	3484	84.0%	273	77.6%
**Population Viral Suppression**	NA	NA	Sampled from *PC*^b^	54.4%	Sampled from *PC*^b^	58.8	3484/8305	42.0%	273/1159	23.6%

^a^The number of residents interviewed exceeded the estimated total residents in the communities by 2542, likely due to population growth over the course of the study

^b^Proportions derived from random sample of 75 individuals from the Population Cohort (PC) of each intervention community, weighted according to the age-sex distribution of the HIV + population; applying PC HIV prevalence estimates to the final intervention data on age/sex structure of the population

In PopART through household enumeration and extrapolation to households that were inhabited but could not be enumerated, 125,911 study eligible individuals were estimated to reside in the four Arm-A intervention communities and 134,839 individuals in four Arm-B communities at baseline and 134,162 (Arm-A) and 142,250 (Arm-B) at endline (Tables 2, 3 and Fig. 1a, b). A total of 83,487 individuals in Arm-A and 82,269 in Arm-B had HIV status ascertained and approximately 17,686 HIV-positive individuals were estimated to reside in the Arm-A communities and 18,274 individuals in Arm-B communities, at baseline. At endline, 87,762 individuals in Arm-A and 85,333 in Arm-B had HIV status ascertained and approximately 17,662 HIV-positive individuals were estimated to reside in the Arm-A communities and 20,541 individuals in Arm-B communities.

**Table 3:** Endline total population 90–90-90 coverage estimates

	**BCPP**		**PopART**				**SEARCH**		**TasP**	
	N	%	A (N)	A (%)	B (N)	B (%)	N	%	N	%
**Estimated total population**	(61,544) 64,086	100%	134,162	100%	142,250	100%	99,186	100%	4290	100%
**Total number with HIV status ascertained** **(+ ve and -ve)**	61,655	96.2%	87,762	65.4%	85,333	60.0%	80,390	81.0%	4097	95.5%
**Estimated total HIV + **	14,270	23.1%	17,662	20.1%	20,541	24.1%	8399	8.5%	1283	29.9%
**Knowledge of HIV + (First 90)**	13,328	93.4%	16,207	91.8%	18,320	89.2%	7759	92.4%	1206	94.0%
**On ART (Second 90)**	12,259/ 13124^a^	93.4%	12,553/ 14127^b^	88.9%	13,784/ 15904^b^	86.7%	7367	94.9%	782^b^	64.8%
**Virally suppressed (3**^**rd**^** 90)**	11,687 /11954^c^	97.8%	Sampled from PC^d^	93.0%	Sampled from PC^d^	89.2%	6612	89.8%	706	90.3%
**Population Viral Suppression**	11,687/ 14,270	81.9%	Sampled from PC^d^	70.2%	Sampled from PC^d^	70.4%	6612 /8399	78.7%	706/1283	55.0%

^a^Denominator reflects people alive at study end

^b^Denominator reflects age eligible people alive at study end and still resident in same area of the community and in-migrants

^c^Denominator reflects people alive at study end and have viral load result

^d^Proportions derived from random sample of 75 individuals from the Population Cohort (PC) of each intervention community, weighted according to the age-sex distribution of the HIV + population; applying PC HIV prevalence estimates to the final intervention data on age/sex structure of the population

In SEARCH, through household census enumeration, 79,818 community residents aged ≥ 15 years were estimated to reside in the 16 intervention communities at baseline and 99,186 at endline. Of these, 71,402 had their HIV status directly measured at baseline and 80,390 at endline. At baseline, an estimated 8,305 PLHIV were estimated to reside in the SEARCH intervention communities, increasing to 8,399 at endline.

In TasP, 4,681 individuals were enumerated in the five TasP communities included in this analysis at baseline and 4,290 individuals at endline, with HIV status ascertained in 4024 and 4097 individuals at baseline and endline, respectively. At baseline, 1,159 PLHIV were estimated to be residing in the relevant communities and this increased to 1,283 at endline.

### Knowledge of HIV-positive status

**Fig. 2:**
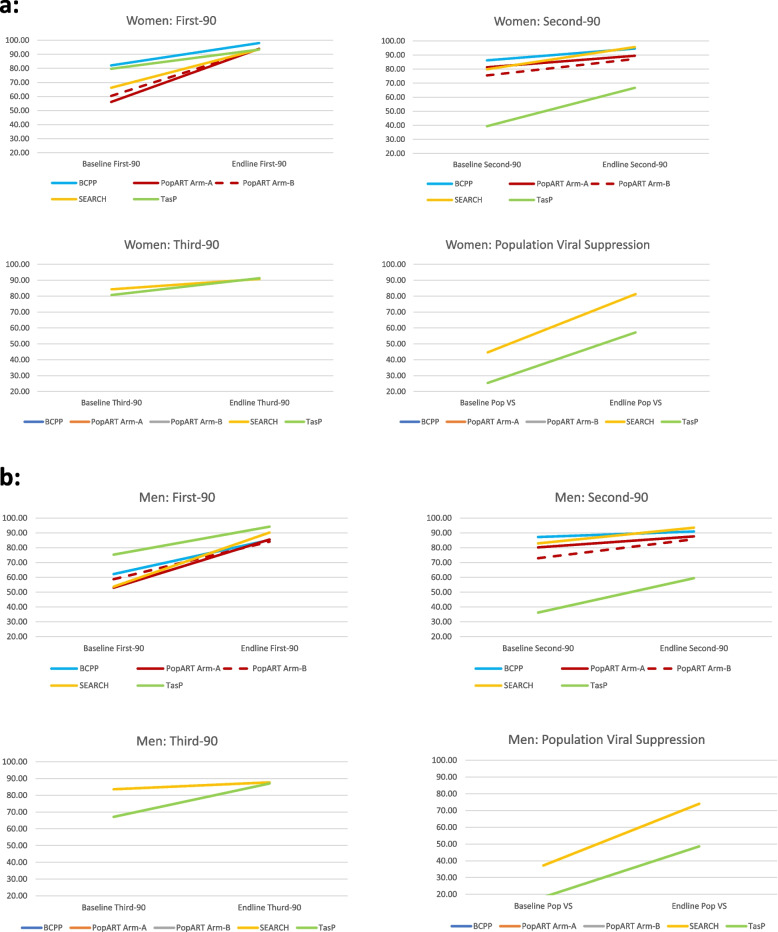
**a** Difference made to 90–90-90 coverage and population viral suppression estimates from baseline to endline among women. **b** Difference made to 90–90-90 coverage and population viral suppression estimates from baseline to endline among men

In BCPP at baseline, before study interventions were implemented, 10,703 individuals had knowledge of a positive HIV status, 75.0% of the estimated HIV positive individuals residing in the community; 82.1% of estimated HIV positive women and 62.2% of estimated HIV-positive men had knowledge of their status (Fig. 2a and b). The number of HIV-positive individuals who knew their status increased to 13,328 by study end (endline), 93% of all estimated PLHIV, (98.0% of the estimated HIV-positive women and 85.2% of estimated HIV-positive men).

In PopART Arm-A at baseline, 9,648 individuals were estimated to have knowledge of HIV-positive status, representing 54.6% of PLHIV (56.1% of HIV-positive women and 53.0% HIV-positive men). By endline, this increased to 16,207 PLHIV or 91.8% of PLHIV (93.9% of women and 85.5% of men). In PopART Arm-B at baseline, 10,877 individuals self-reported knowledge of a positive HIV status, 59.5% of PLHIV (60.4% of HIV-positive women and 58.7% HIV-positive men). By endline this increased to 18,320 PLHIV or 89.2% of all PLHIV (93.8% of women and 84.1% of men).

Baseline knowledge of HIV-positive status was similar in SEARCH. An estimated 5,136 PLWHIV knew their status at baseline, 61.8% of all PLHIV (66.2% of women and 53.7% of men). This increased to 92.4% at endline (prior to the final delivery of trial intervention services in SEARCH) (*N* = 7,759 PLHIV with known status; 93.5% of women and 90.2% of men).

Baseline knowledge of HIV-positive status was highest in TasP at 78.6% (*N* = 911) (79.7% of women and 75.3% of men). Endline knowledge of HIV-positive status was also the highest in TasP among the trials at 94.0% (*N* = 1206) (93.4% of women and 94.2% of men).

### PLHIV on ART

Of the 10,703 HIV-positive individuals who knew their status before the study started in BCPP, 9,258 (87%) were on ART (86% of diagnosed HIV-positive women and 87% of diagnosed HIV-positive men) – the highest proportion on ART at baseline among the trials. Of the 13,328 HIV-positive individuals identified by BCPP throughout the study, 204 died by study end. Of the remaining 13,124 individuals, 12,259 (93%) were on ART (95% of diagnosed HIV-positive women and 91% diagnosed HIV-positive men).

At baseline in PopART Arm-A communities, 7,805 of 9,648 diagnosed PLHIV were on ART (80.9% overall; 81.3% women and 80.2% men), while in Arm-B the proportion was slightly lower with 8,112 of 10,877 on ART (74.6% overall; 75.5% women and 72.9% men). At endline, 12,553 of 14,127 diagnosed PLHIV were on ART in Arm-A, an increase to 88.9% overall (89.5% women and 87.6% men), while in Arm-B the increase compared to baseline was higher than in Arm-A with 13,784 of 15,904 on ART by the end of the trial (86.7% overall; 87.2% women and 85.7% men).

In SEARCH, an estimated 4,149 of 5,136 diagnosed PLHIV were on ART at baseline (80.8% overall; 79.9% women and 82.9% men), while at endline, this increased to 7,367 on ART of 7,759 diagnosed PLHIV (94.9% overall; 95.7% women and 93.5% men).

In TasP, a modest 38.6% (*N* = 352) of 911 diagnosed PLHIV were on ART at baseline (39.4% women and 36.2% men). The proportion on ART by endline increased more in TasP than other trials; however absolute coverage was lower; of 1,206 diagnosed PLHIV, 782 were on ART (64.8% overall; 66.7% women and 59.4% men), by endline.

### Viral suppression

Baseline viral suppression metrics are not available for BCPP. Among the 11,954 individuals on ART with viral load results available at endline in BCPP, 11,687 (97.8%) were virally suppressed. With a conservative assumption that the 305 (2.5%) individuals on ART without a viral load result at endline were not virally suppressed, then the proportion of persons on ART who were virally suppressed dropped to 95.3%. Population level viral suppression at study end (i.e. the number of individuals virally suppressed divided by the estimated number of PLHIV) was 82% (11,687/14,270) overall. For women, population level viral suppression at study end was 88% (8,018/9,167) and 72% for men (3,669/5,103).

PopART viral suppression data were obtained from the *Population Cohort* as described above. At baseline in PopART, the proportion virally suppressed among PLHIV on ART was 88.7% in Arm-A and 91.9% in Arm-B, and at endline was 93.0% in Arm-A and 89.2% in Arm-B. Population viral suppression in PopART was 54.4% (Arm-A) and 58.8% (Arm-B) at baseline, and increased to 70% in both Arms by endline.

In SEARCH at baseline, an estimated 3,484 PLHIV were virally suppressed among 4,419 on ART (84.0% overall; 84.3% women and 83.6% men). By endline, an estimated 6,612 PLHIV were virally suppressed among 7,367 on ART (89.8% overall; 90.8% women and 87.7% men). Population viral suppression in SEARCH increased from 42.0% (44.6% women and 37.2% men) at baseline to 78.7% (81.3% women and 74.0% men) at endline.

In TasP, 273 PLHIV were virally suppressed among 352 on ART at baseline (77.6% overall; 80.7% women and 67.1% men), and by endline 706 PLHIV were virally suppressed among 782 on ART (90.3% overall; 91.3% women and 87.0% men). Population viral suppression in TasP increased from 23.6% (25.4% women and 18.3% men) at baseline to 55.0% (57.2% women and 48.6% men) at endline.

## Discussion

While the trials were ongoing, national policies moved to recommend treatment for all, and leveraging lessons learned from the trials on community-based testing, supported linkage and patient-centred care, the 90–90-90 target has subsequently been reached in Uganda, Kenya and Botswana. Granich et al., [21] All four UTT trials implemented multicomponent interventions that aimed to increase coverage across the HIV care cascade at a population level, with the ultimate goals of reducing HIV incidence and improving community health. Across the trials, interventions included population-level testing to increase knowledge of HIV status among PLHIV, interventions to support linkage to HIV care and rapid ART start following diagnosis, and interventions to support retention in HIV care among persons on ART. The UNAIDS 90–90-90 targets thus provided a useful benchmark for evaluating the effectiveness of intervention deployment on core implementation outcomes, and all trials generated estimates of progress towards these targets. It is acknowledged, however, that achievement of the 90–90-90 targets may still leave subgroups of the population with lower coverage, and some of these may be the very subgroups that contribute disproportionately to onward HIV transmission and sustained HIV-incidence at population level. In settings where HIV is principally concentrated within key populations, the approaches used to deliver universal coverage and to measure coverage against the 90–90-90 targets may be different. Joulaei et al., [22] Our study describes trials conducted in sub-Saharan African settings with generalised HIV epidemics.

The data available, and therefore the estimation approaches employed, differed across the trials. However, in all trials, cascade coverage and overall population level suppression increased substantially over time in the intervention communities.

### Comparative methodology

Each study used methods appropriate for their study design and resulting data structure and estimated the cascade according to their context. There were differences and similarities in the approaches used by the trials to measure 90–90-90 related targets. All the trials except BCPP undertook population-wide censuses at baseline and endline to enumerate the underlying populations of the intervention communities. In contrast, BCPP used a 20% random sample of households to estimate the total population and the same estimate was used in the denominator at endline. All four trials attempted to reach all community residents in order to test and/or identify all PLHIV at both baseline and endline. In SEARCH, TasP and PopART, the results of this population-based testing were also used to estimate HIV prevalence, and by extension total number of PLHIV at baseline and endline. In BCPP, however, HIV prevalence was estimated at baseline from the 20% random sample of households and assumed to be constant at endline.

All the trials measured knowledge of HIV-positive status and ART uptake in their population of interest. PopART was the only trial to rely principally on self-report for these measures, while BCPP, TasP and SEARCH relied principally on electronic health records from clinics or laboratories within their respective countries’ routine health systems (augmented in SEARCH with viral load data, with the assumption that all virally suppressed PLHIV were on ART). Self-report carries an inherent risk of reporting bias, but has the advantage of capturing current information directly from the participant. Electronic health records have the advantage of providing objective proof (such as a documented HIV test result or ART prescription) but are subject to weakness within health systems, such as gaps, delays or errors in electronic data capture.

Viral suppression data were not collected by BCPP at baseline and in PopART viral suppression at baseline and endline was estimated from a research cohort (viral loads from 75 randomly selected PLHIV from *Population Cohorts* of 2000–2500 randomly selected individuals per community) rather than measured directly in the population reached by the intervention. The use of research cohorts in BCPP (for total population and HIV prevalence estimation) and PopART (for viral suppression data) carry some strengths and limitations. Random sampling for research may provide a more representative sample of population viral suppression than reliance of those captured by the intervention data. However, research cohorts consist of those who consent to research participation and may be systematically different from the general population in terms of health behaviours. The composition of those who can be recruited may be different. For instance, in cohorts recruited at home the proportion of women is likely to be higher than men and this was indeed observed in the UTT trials. While statistical adjustments were made to account for gender imbalances the potential for bias remains. Further, research participants are subject to the Hawthorne effect – a phenomenon whereby the very awareness of being observed alters behaviour. Relatedly, the behaviours of those who have regular contact with researchers may be altered by the support received, for instance with benefits to treatment adherence. Further, research procedures can distort outcomes, for example in the PopART a rapid HIV test was offered at each *Population Cohort* survey (and those diagnosed HIV-positive referred for care).

The use of process data obtained through intervention delivery has greater scope to be representative of the general population, or at least the members of the population who engage with health services. In particular, PLHIV not engaged in HIV care are at much higher risk of non-suppression. For this reason, both TasP and BCPP conservatively assumed that persons without a viral load on record were unsuppressed. In contrast, SEARCH leveraged population-level testing with the aim of collecting viral loads on all PLHIV outside the routine care setting. A hybrid model of multi-disease health fairs, with subsequent tracing and offering of services at home or other location for persons not attending the health fair, was used to maximize coverage of viral load measurements. Estimates of suppression were further adjusted for baseline demographics and care history when extrapolating to the total population.

While all the trials used pre-intervention measures and estimates to represent baseline data, SEARCH was the only trial to use data collected prior to the final round of intervention delivery to represent endline data. This is an important consideration, especially when comparing knowledge of status among PLHIV (first-90) across trials, as estimates will be higher when including services offered during the final delivery of the intervention. Other factors which are relevant when interpreting and comparing estimates include the size of the communities intervened in—larger communities pose greater challenges for complete coverage; the duration of intervention delivery—the longer the intervention the more likely the intervention is to be fully embraced by the communities, although “fatigue” may set in over time; and the level of population movement in and out of the trial communities – mobile communities are harder to represent through intervention or process data. SEARCH, TasP and PopART communities involved highly mobile populations and faced the associated challenges of capturing a fluctuating picture [17, 23].

### Generalisability of the trials’ approaches to measurement of 90–90-90 coverage

The methods used by the respective trials presented in this paper highlight the scope for different approaches to measure coverage. All the UTT trials had the same goals to maximise coverage of HIV services across the care cascade, but there were subtle differences which were largely influenced by the pre-existing contexts against which the trials were established. For instance, in settings where government electronic health records were fairly comprehensive, they were utilised to measure coverage provided by local health services, but if such systems were not in place, self-report was used allowing for capture of care received in the private sector or distant health centres. This study has not uncovered any one approach which is superior, rather that several approaches are available and researchers or policy makers seeking to measure coverage should utilise what is best suited for their contexts.

### Findings

#### First-90 – knowledge of HIV-positive status

Baseline knowledge of HIV-positive status was notably different in the respective trial communities with the lowest proportions previously diagnosed with HIV in the PopART communities (54.6% and 59.5% in PopART Arm-A and Arm- B, respectively) and the highest proportion in TasP communities (78.6%). All the trials achieved similar coverage in knowledge of HIV-positive status among PLHIV by endline (89.2% – 94.0%), with the highest proportion achieved in TasP (94.0%) and the greatest increase in PopART Arm-A from 54.6% at baseline to 91.8% at endline. The direct comparison with SEARCH is limited by the fact that their endline estimates represent knowledge of HIV status prior to endline intervention delivery.

Knowledge of HIV-positive status at baseline was consistently higher among women than men with the most substantial gender differences seen in BCPP, where the proportion was 82.1% among women but just 62.2% among men. By endline, differences between men and women were much smaller across all the trials, mostly with less than 10% difference although the gap remained highest in BCPP (98.0% women and 85.2% men). In general, the universal testing methods used by all four trials made a greater difference to knowledge of HIV-positive status for men than women, except in PopART Arm-A where the largest baseline-to-endline difference was achieved overall but more so among women than men (37.8% increase from 56.1% to 93.9% among women vs 32.5% increase from 53.0% to 85.5% among men). The SEARCH trial achieved the largest gain among men, with knowledge of HIV-positive status increasing by 36.5% from 53.7% prior to baseline intervention to 90.2% prior to the endline intervention.

#### Second-90—PLHIV on ART

All the trials except TasP had fairly high uptake of ART among diagnosed PLHIV at baseline (74.6% – 86.5%) and achieved increases of ~ 10% by endline (86.7% – 94.9%). The situation in TasP was the exception, with baseline uptake of ART at just 38.6%. Despite a substantial increase by 26.2%, endline ART coverage remained substantially below second-90 targets at 64.8%. Challenges faced in achieving linkage to care in order to initiate ART have been reflected on elsewhere [24–26]. In summary, stigma was an overarching barrier to ART uptake in the TasP communities at the individual (fear of HIV disclosure, “fear of being seen”), community (community religious perceptions) and health system levels (standalone clinics for only HIV positive participants). Other barriers included alternative health beliefs and fear of ART side effects [24, 27, 28].

Gender differences within trials with regard to uptake of ART at baseline were minimal (~ 3% or less) and increases by gender were also of similar magnitude (~ 5% or less difference between genders) within trials. The smallest increase was among men in BCPP (from 87.2% at baseline to 90.9% at endline), although both proportions were high and close to the 90% target. The greatest baseline-to-endline increase was among women in TasP (from 39.4% at baseline to 66.7% at endline).

#### Third-90 – viral suppression on ART

Baseline vs endline comparisons for viral suppression among PLHIV on ART are not available for BCPP, but endline proportions were very high at 95–98% depending on assumptions about those among whom data were not obtained. Similarly, viral suppression in PopART, based on measures among a small random sample of ~ 75 individuals from each trial community, were already around the 90% target at baseline in both Arm-A and Arm-B. Increases were achieved by endline in Arm-A from 88.7% at baseline to 93.0% at endline. In Arm-B, a small baseline-to-endline reduction in viral suppression was observed at baseline from 91.9% to 89.2% at endline; however, this minor difference more likely represents unchanged levels of viral suppression among those on ART which started high and remained so at endline. In SEARCH, there was an increase in viral suppression among those on ART from 84.0% prior to the baseline intervention to 89.8% prior to the endline intervention. The greatest baseline-to-endline increase in viral suppression was achieved in TasP (12.7% increase) from the lowest among the trials with baseline data, with 77.6% viral suppression at baseline and well below the third-90 target, to 90.3% by endline.

The endline proportions of those virally suppressed among PLHIV on ART in BCPP were nearly identical between men and women, while PopART gender-stratified data are limited by the small number of men in the sample of ~ 75 per community with viral loads. SEARCH found a slightly greater increase in viral suppression among women than men (6.5% among women vs 4.1% among men). There were notable increases for both genders in TasP but the increase was greatest among men (increase of 19.9% from 67.1% at baseline to 87.0% at endline among men vs 80.7% at baseline to 91.3% at endline among women).

#### Population viral suppression

Population viral suppression provides an estimate of viral suppression among all PLHIV in the trial communities, irrespective of whether HIV was diagnosed or ART initiated, and is the overall outcome of the individual steps in the cascade of care which are measured against the 90–90-90 targets. If the targets are satisfied, population viral suppression of 72.9% or greater would be achieved. BCPP and SEARCH exceeded the 72.9% target (81.8% and 78.7% respectively) with SEARCH achieving the largest baseline-to-endline increase (36.8% increase). Endline population viral suppression in both PopART intervention arms were very close to the 72.9% target (both ~ 70%) but increases from baseline were the smallest (increase from baseline of 15.8% in Arm-A and 11.6% in Arm-B). Baseline population viral suppression was just 23.6% in TasP and by endline this was more than doubled to 55.0%, but still well below the 72.9% target. The inability to achieve adequate linkage into care and therefore ART initiation meant that viral suppression among all PLHIV in the community remained low in TasP.

Interpretation of coverage data presented in relation to the 90–90-90 targets which involves changes over time, require due consideration of the fact that the measures are based on cross-sectional estimates at different time-points. For instance, the proportions presented for the second-90 target appear unchanged or even diminished at endline compared to baseline. The underlying reason for this is that the denominator (the number of diagnosed PLHIV) was substantially larger at endline due to the success of universal testing in the trials. Additionally, as it may take individuals time to initiate ART following diagnosis, the numerator for the second-90 may take longer to change. It has been suggested that the 90–90-90 targets may lead to incorrect conclusions and longitudinal metrics should be included wherever possible [29]. Indeed, each of the trials have previously published such analyses [10, 11, 13, 14, 18, 20, 26, 30]. Further, the denominator of the first-90 (the total number of people living with HIV population) relies on proxy measures that are estimated with assumptions and extrapolation, and inaccuracies at this step could result in misleading estimates at later steps.

Results from the TasP trial reveal that increasing knowledge of HIV-positive status alone will not impact the whole cascade if every step cannot be similarly boosted. The second-90 target encompasses both the need for a high enough proportion of individuals to link into care, and once linked for those individuals to accept and initiate treatment. Existing evidence suggests that the former poses the greater challenge and once linked, patients are willing to initiate ART [31–33]. Linkage is thought to be directly connected to motivation to initiate ART; however, there are no established targets against which to compare the component steps and direct comparison of the two steps is limited. Viral suppression among those on ART appears to be consistently high in sub-Saharan settings [34] and the data from the UTT trials support this.

## Conclusion

This study has not uncovered any one estimation approach which is superior, rather that several approaches are available and researchers or policy makers seeking to measure coverage should reflect on background contexts and complexities that affect estimation of population-level coverage in their specific settings. The evidence summarised here illustrates that all four UTT trials surpassed UNAIDS targets for universal testing in their intervention communities ahead of the 2020 milestone. This arguably represents one of the greatest public health achievements of the trials, and was translated to substantial benefits in terms of enabling undiagnosed PLHIV (as well as PLHIV previously diagnosed but out-of-care) to access treatment earlier for their own benefit and to prevent onward transmission. All but one of the trials also achieved the 90–90 targets for treatment and viral suppression. It is beyond the scope of this paper to examine costs of intervention delivery but cost-effectiveness analyses suggest that in the long-term UTT is worthwhile [35–37]. UTT creates improvements in all the steps of the cascade of care when compared to baseline levels in a range of contexts examined here and remains a realistic option to achieve 95–95-95 by 2030 and fast-track the end of the epidemic.

## Supplementary Information

**Additional file 1: Supplementary Figure 1.** UNAIDS 90-90-90 targets for HIV treatment coverage. **Supplementary Table 1.** Sources of data for measuring 90-90-90 targets in individuals receiving trial interventions.

## Data Availability

All data generated or analysed during this study are included in this published article [and its supplementary information files].
