# Supplementary material for: Achieving the UNAIDS 90–90-90 targets: a comparative analysis of four large community randomised trials delivering universal testing and treatment to reduce HIV transmission in sub-Saharan Africa
Source: BMC Public Health. 2022 Dec 13;22:2333. doi: 10.1186/s12889-022-14713-5 (PMC9746009; doi:10.1186/s12889-022-14713-5)
Supplement: Supplementary file 1 — Additional file 1: Supplementary Figure 1. UNAIDS 90-90-90 targets for HIV treatment coverage. Supplementary Table 1. Sources of data for measuring 90-90-90 targets in individuals receiving trial interventions. [file 12889_2022_14713_MOESM1_ESM.docx]

Supplementary Figure 1: UNAIDS 90-90-90 targets for HIV treatment coverage
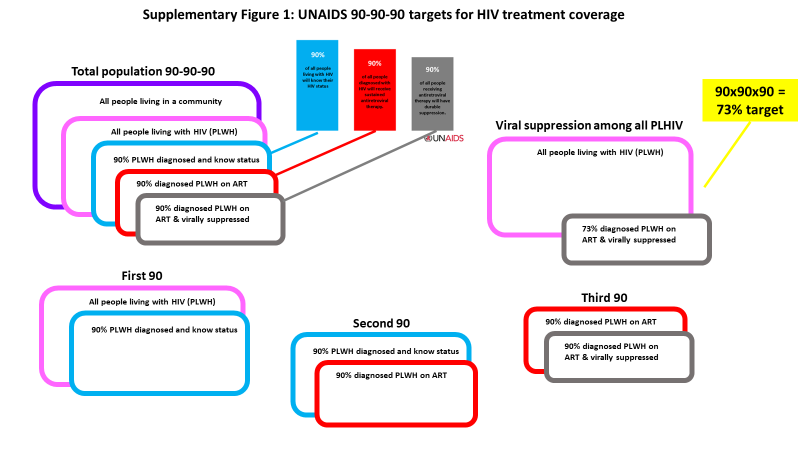


**Supplementary Table 1: Sources of data for measuring 90-90-90 targets in individuals receiving trial interventions**

|  | **BCPP** | **PopART** | **SEARCH*** | **ANRS 12249 TasP** |
| --- | --- | --- | --- | --- |
| **Baseline first-90** | | | | |
| Source of observed data on number HIV+ve **(denominator 1^st^ 90)** | - Not measured directly – estimated (summarised in Table 2) | Data collected among population reached by the intervention at the household   - HIV antibody POC test result - Self-report HIV+ve - See below re those who didn’t self-report HIV+, didn’t accept offer of testing, and didn’t report testing in the previous 3 months | Data collected among population reached at baseline health fair   - HIV antibody POC test result - Detectable HIV RNA - MoH record of HIV care PRIOR to baseline testing | Data collected among population reached by the intervention at the household   - HIV antibody POC test result - Self-report HIV+ve - HIV clinic visits in local primary healthcare clinics ** - DBS blood samples collected at home (Elisa assay) |
| Source of observed data on number with knowledge of HIV+ve status **(numerator 1^st^ 90)** | Documentation of a prior HIV+ test or on ART status prior to start of testing campaigns. | - Self-reported prior knowledge of HIV+ve status among population reached by the intervention at baseline | - MoH record of HIV care PRIOR to baseline testing or an undetectable VL measured at baseline | - Self-reported prior knowledge of HIV+ve status among population reached by the intervention at baseline - Routine electronic clinical care record of prior positive HIV rapid test, HIV care or ART uptake among population reached by the intervention at baseline |
| **Endline first-90** | | | | |
| Source of observed data on HIV status for number HIV+ve **(denominator 1^st^ 90)** | - Same as baseline | - Analogous to baseline, time-updated at endline | - Analogous to baseline, time-updated at endline | - Analogous to baseline, time-updated at endline - Includes estimated unobserved seroconverters (see Table 2 assumptions) |
| Source of observed data on number with knowledge of HIV+ve status **(numerator 1^st^ 90)** | - Analogous to baseline PLUS those who tested HIV+ as part of endline intervention home visit or in mobile settings in the community during the testing campaigns. | - Self-report HIV-positive among population reached by the intervention at baseline PLUS those who tested HIV+ as part of endline intervention home visit | - Analogous to baseline, time updated for diagnosis PRIOR to endline (i.e. does not include uptake of testing offered at endline health fair) | - Analogous to baseline, time-updated at endline PLUS those who tested HIV+ as part of endline home visit |
| **Baseline second-90** | | | | |
| Source of observed data on number with knowledge of HIV+ve status **(denominator 2nd 90)** | - As for numerator of baseline 1^st^ 90 | - As for numerator of baseline 1^st^ 90 | - As for numerator of baseline 1^st^ 90 | - As for numerator of baseline 1^st^ 90 |
| Source of data for  number with knowledge of HIV+ve status and on ART **(numerator 2^nd^ 90)** | - Documentation of being on ART (health cards, prescriptions, and/or pill bottles) at intake or was later retrieved from routine electronic clinical care record if the individual did not have documentation at intake | - ART initiation prior to baseline as evidenced by self-report at first participation among population reached by the intervention at baseline | - MoH record of ART initiation PRIOR to baseline testing or an undetectable VL measured at baseline health fair | - ART prescription in trial or MoH clinic in the last 3 months prior to baseline intervention visit date - An undetectable VL in the 13 months prior to baseline HH visit date (even if ART not documented) in routine electronic clinical care records |
| **Endline second-90** | | | | |
| Source of observed data on number with knowledge of HIV+ve status **(denominator 2nd 90)** | - Total number of HIV+ve who knew their status and were alive at endline. | - As for numerator of endline 1^st^ 90 | - As for numerator of endline 1^st^ 90 | - As for numerator of endline 1^st^ 90 |
| Source of data for  number with knowledge of HIV+ve status and on ART **(numerator 2^nd^ 90)** | - Number with electronic record of ART refill within previous 4m or clinic appointment within the previous 6 months after a prior ART initiation date or status as “on HAART/on therapy” in the electroni c record - VL result in the previous 18 months after initiation was also evidence of retention on ART** | - Number on ART self-reported at the last follow-up visit made during the endline intervention delivery, among population reached by intervention at endline | - Analogous to baseline, time-updated to reflect status prior to endline | - Analogous to baseline, (time-updated upto last home visit of endline intervention round)  - ART initiation in trial or MoH clinic within 3m after last home visit was additionally included |
| **Baseline 3^rd^ 90** | | | | |
| Source of observed data on number with knowledge of HIV-positive status and on ART **(denominator 3rd 90)** | - NA – see below re numerator 3^rd^ 90 | - PC cohort members who were enrolled at PC0 and self-reported they were on ART, among whom a random sample had viral load testing done | - As for numerator of baseline 2^nd^ 90 | - As for numerator of baseline 2^nd^ 90 |
| Source of data for  number with knowledge of HIV+ve status and on ART who are virally suppressed **(numerator 3^rd^ 90)** | - Viral loads were not collected at time of intake in the intervention communities and no estimates are available | - Viral load testing on a random sub-sample of PC0 members who were confirmed HIV-positive (based on laboratory testing of a venous blood sample) | - Viral loads measured at baseline health fair | - All VL data from trial clinics or routine electronic clinical care records - Linear interpolation performed between time points, suppression defined as VL < 400 - If no VL prior to t, the individual was considered as not having reached the 3^rd^ 90. - If no VL posterior to t, the value of the last known VL was used. |
| Endline 3^rd^ 90 | | | | |
| Source of observed data on number with knowledge of HIV+ve status **(denominator 3rd 90)** | - Number of HIV positive persons identified through BCPP on ART, were alive at study end, and had an available VL result | - PC36 cohort members who self-reported they were on ART, among whom a random sample had viral load testing done | - As for numerator of endline 2^nd^ 90 | - As for numerator of endline 2^nd^ 90 |
| Source of data for  number with knowledge of HIV+ve status and on ART who are virally suppressed **(numerator 3^rd^ 90)** | - Last VL test result from January 2017 through June 2018 in the routine electronic clinical care record or collected by BCPP in the community. | -Viral load testing on a random sub-sample of PC36 members who were confirmed HIV-positive (based on laboratory testing of a venous blood sample) | Analogous to baseline, time-updated to endline | Analogous to baseline, (time-updated upto last home visit of endline intervention round)  Undetectable VL within 3m after last home visit was additionally included |

*Throughout for SEARCH study, ‘health fairs’ refers to multidisease testing conducted in each community through two-week, out-of-facility health fairs with follow-up for non-attendees at a location of their choice (usually home).

**The majority of data were from within the previous 6 months but up to 18 months was included to allow for limitations in routine data.
